# Implementation of SARS-CoV-2 Wastewater Surveillance Systems in Germany—Pilot Study in the Federal State of Thuringia

**DOI:** 10.3390/microorganisms14020277

**Published:** 2026-01-24

**Authors:** Felix Kaller, Gloria M. Kohlhepp, Sarah Haeusser, Sara Wullenkord, Katarina Reichel-Kühl, Anna Pfannstiel, Robert Möller, Jennifer Führ, Carlos Chillon Geck, Yousuf Al-Hakim, Andrea Lück, Norbert Kreuzinger, Johannes Pinnekamp, Mathias W. Pletz, Claudia Klümper, Silvio Beier, Kay Smarsly

**Affiliations:** 1 Department Hamm 2, Hamm-Lippstadt University of Applied Sciences, 59063 Hamm, Germany; 2 Bauhaus-Institute for Infrastructure Solutions (b.is), Bauhaus University Weimar, 99423 Weimar, Germany; 3 Analytik Jena GmbH + Co. KG, 07745 Jena, Germany; 4 Institute of Digital and Autonomous Construction, Hamburg University of Technology, 21079 Hamburg, Germanyyousuf.al-hakim@hsu-hh.de (Y.A.-H.); kay.smarsly@tuhh.de (K.S.); 5 Institute for Water Quality and Resources Management, Vienna University of Technology, 1040 Vienna, Austria; 6 Institute of Environmental Engineering, RWTH Aachen University, 52074 Aachen, Germany; 7 Institute for Infectious Diseases and Infection Control, Jena University Hospital—Friedrich Schiller University Jena, Am Klinikum 1, 07740 Jena, Germany; mathias.pletz@med.uni-jena.de

**Keywords:** public health, SARS-CoV-2, wastewater monitoring, wastewater surveillance systems, early warning systems, epidemiological data integration, process optimization, institutionalization, data interoperability

## Abstract

Since the COVID-19 pandemic, wastewater monitoring has become an additional tool in the surveillance of infectious diseases. Many EU countries put wastewater surveillance systems (WSS) in place to track SARS-CoV-2 and its variants and other pathogens, such as the influenza virus or Respiratory syncytial virus (RSV). In Germany, several research and pilot projects funded by the EU, the Federal Ministry of Education and Research, the Federal Ministry of Health, and projects at Federal State level have been launched in the last four years. In Germany, wastewater monitoring was not implemented as a public health tool before the COVID-19 pandemic, but in September 2022, it has been legally determined in the German infection protection act (Infektionsschutzgesetz, IfSG). As Germany is a federal state, competencies in epidemic management partly belong to the 16 federal states (“Länder”). In the federal states, the local health authorities at the county (“Kreise”) level also have specific risk management and communication competencies. Furthermore, WSS has been incorporated into the revised Urban Wastewater Treatment Directive (EU) 2024/3019. For this reason, the federal states and local health authorities play a pivotal role in successfully implementing wastewater monitoring as a supplementary component of disease surveillance in Germany. Between November 2021 and August 2022, the federal state of Thuringia, Germany, supported a pilot study to implement a surveillance system for SARS-CoV-2-RNA in wastewater of 23 wastewater treatment plants in 17 counties in Thuringia. Here, we describe the study design and the system behind the logistics and the planning, and we provide an overview of the options for involving the public health service. Furthermore, the possibilities for IT concepts and approaches to innovative AI solutions are shown. We also aim to explore the feasibility and potential barriers to further implementing wastewater surveillance as a supplementary public health tool in Thuringia.

## 1. Introduction

Risk management in the coronavirus disease 2019 (COVID-19) pandemic has been a challenge for public health authorities worldwide [1]. Measures to control virus transmission and protect human health must be effective and adequate. In this context, high-quality and accurate surveillance data are mandatory for informed decision-making in pandemic management. Traditional epidemiological data, such as reported cumulative COVID-19 incidence, COVID-19-related hospitalization, and death rates, play a crucial role in surveillance systems. However, in a pandemic situation, data quality is always an issue of concern, and several factors, such as the applied testing strategy and reporting delays, compromise the informative value of the data [2].

As virus are excreted via feces by infected individuals, monitoring SARS-CoV-2 RNA in wastewater is a complementary surveillance approach. In March 2021, the EU Commission adopted a recommendation on a common approach to establish systematic surveillance of SARS-CoV-2 and its variants in wastewater in the EU [3]. Following this recommendation, many EU countries put in place wastewater monitoring systems to track SARS-CoV-2 and its variants [4] as well as other pathogens, such as the influenza virus or RSV [5,6,7,8]. In Germany, several research and pilot projects funded by the EU, the Federal Ministry of Education and Research, and the Federal Ministry of Health as well as projects at the Federal State level, have been launched in the last four years [4,9,10,11,12]. However, most studies so far investigated technical and analytical issues concerning the detection of SARS-CoV-2 RNA or its variants in wastewater and the correlation between virus load in wastewater and reported numbers of SARS-CoV-2 positive cases [13,14,15].

Currently, only a few studies describe the needs and perspectives of local health authorities regarding the practical implementation of wastewater surveillance [16,17]. A survey of public health agencies in the United States showed that despite the growing interest and confidence in the effectiveness of wastewater monitoring, there are still limiting factors preventing an expanded implementation in the public health sector [16]. In Germany, wastewater monitoring was not implemented as a public health tool before the COVID-19 pandemic. Since September 2022, wastewater monitoring has been legally determined in the German infection protection act (Infektionsschutzgesetz, IfSG) as an indicator for assessing the epidemiologic situation. As Germany is a federal state, competencies in epidemic management partly belong to the 16 federal states (“Länder”). In the federal states, the local health authorities at the county (“Kreise”) level have specific responsibilities in managing risks and communicating effectively. For this reason, the federal states and local health authorities play a pivotal role in successfully implementing wastewater monitoring as a supplementary component of disease surveillance in Germany.

Between November 2021 and August 2022, the federal state of Thuringia, Germany, supported a pilot study to implement a surveillance system for SARS-CoV-2-RNA in wastewater of 23 wastewater treatment plants (WWTP) in 17 counties in Thuringia. This pilot study was referred to as the SARS-**Co**V-2 wastewater **mo**nitoring in **Th**uringia (CoMoTH) project. Results on the quantitative correlation between data from wastewater monitoring and clinical indicators have already been published and discussed in Haeusser et al. [18]. Here, we outline the research design, logistics, and planning involved. We also examine the challenges crucial for local health authorities’ engagement. Additionally, we present possibilities for IT concepts and innovative AI solutions that could enhance our approach. We aim to explore the feasibility of implementing wastewater surveillance as a supplementary public health tool in Thuringia while also identifying potential barriers to its further adoption.

## 2. Methods—Study Design, Framework and Stakeholders

Stakeholders on a regional and local level play a crucial role in the effective implementation of wastewater surveillance systems. In Germany, many responsibilities for surveillance and monitoring are assigned to the federal states or even local authorities. Therefore, the study design was developed based on the existing framework conditions and the involvement of stakeholders in Thuringia.

The development of a wastewater monitoring concept customized for Thuringia’s specific regional conditions has to address local challenges effectively. An effective approach has to consider (1) water management characteristics, (2) structural aspects of public health, and (3) analytics.

**Figure 1:**
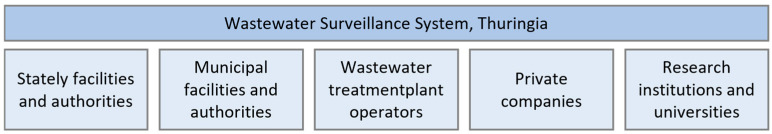
Wastewater surveillance systems—stakeholders.

Figure 1 shows Thuringia’s most important stakeholders for implementing a wastewater surveillance system (WSS). The state-level authorities, such as the Thuringian State Office for Consumer Protection (TLV), and the municipal facilities, such as health authorities, hold administrative responsibilities. WWTP—operators and private companies oversee the plant operations and monitoring of wastewater quality. Research institutes and universities are focused on developing and improving analytical and data science methods.

### 2.1. Wastewater Treatment Plants Involved in the Study

The criteria for selecting the wastewater treatment plants (WWTPs) included size classification, the proportion of industrial wastewater, knowledge of the sewer networks connected to vulnerable and medical facilities, and the presence of tourist attractions.

**Figure 2:**
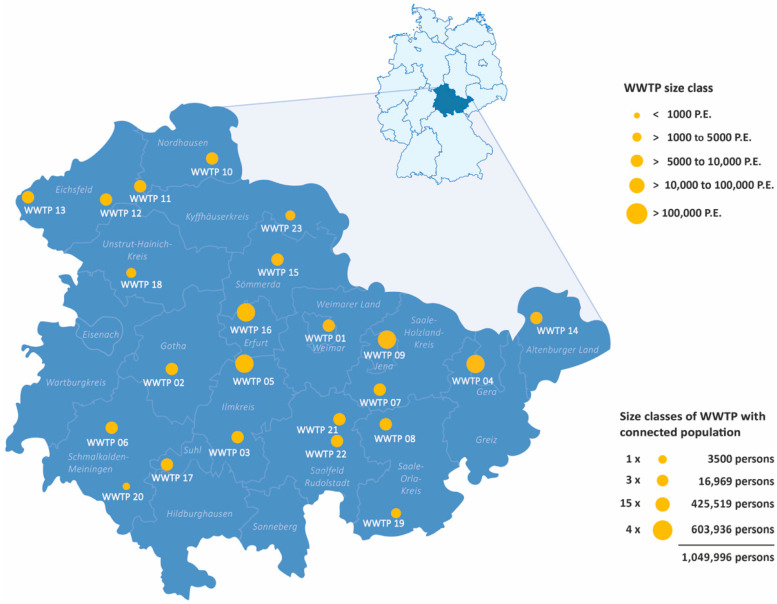
The 23 WWTPs in Thuringia involved in the project; P.E.: population equivalent.

The graphic below shows the wastewater treatment plants (WWTP) in Thuringia that were part of the project. The study involved 23 WWTPs across 17 counties in Thuringia, with a total of between 3500 and 317,274 connected inhabitants. (see Figure 2). In Germany wastewater treatment plants are categorized into size classes based on the number of population equivalents (P.E.) they are designed to treat. A population equivalent is a standardized measure to compare the pollution load from households and industry [19,20]. It refers to the amount of organic biodegradable load having a five-day biochemical oxygen demand (BOD_5_) of 60 g of oxygen per day, corresponding to the average contribution of one person, as defined in Appendix A of the German Wastewater Ordinance, which applies to wastewater treatment plants in Germany [21].

Detailed information on the selected WWTPs can be found in the supplement (see Appendix A, Table A1).

### 2.2. Health Authorities Involved—Stately and Local Level

The Free State of Thuringia comprises 22 local health authorities, six of which are affiliated with independent cities: Weimar, Jena, Erfurt, Gera, Suhl, and Eisenach. The State Administration Office (Thüringer Landesverwaltungsamt (TLVwA)) technically supervises the health authorities. Additionally, general tasks for preventing and controlling communicable diseases are managed at the state level.

**Figure 3:**
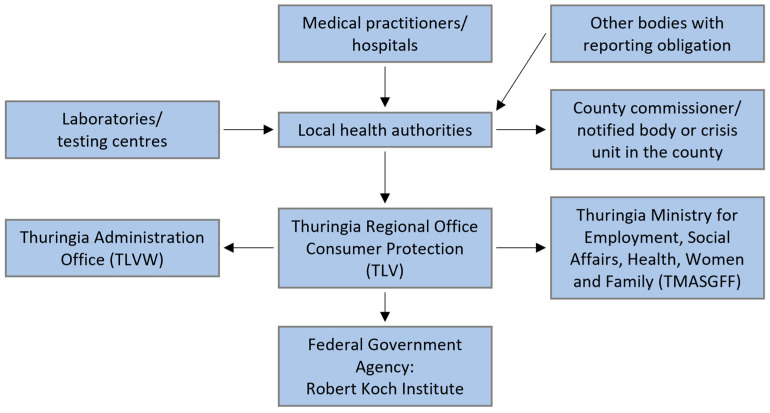
Information and reporting channels for the Free State of Thuringia.

Figure 3 shows the infection control reporting system in Thuringia. At the time of this study the following reportable case definition applied according to the the German infection protection law a suspected illness, illness, and death concerning COVID-19 and direct or indirect evidence of pathogen detection of SARS-CoV-2 is reported to the local health authority. The encrypted reports are then retrieved, checked, and further processed using specific reporting software (German Electronic Reporting and Information System for Infection Protection, DEMIS 1.0—as of 2021/2022) at the health department. Insofar as the case definition specified by the German National Public Health Institute (Robert-Koch-Institute, RKI) is met, the health authorities transmit the data to the responsible state authority in the Thuringia Regional Office of Consumer Protection (Thüringer Landesamt für Verbraucherschutz (TVL)), which then sends the data to the RKI.

In addition, other information and exchange channels exist in the context of risk management in the event of a pandemic, (1) at the districts and cities level with the district administrators/mayors or the local crisis team and (2) at the state level via the TVL with the TLVwA and the Thuringian Ministry of Social Affairs, Health, Labour and Family (Thüringer Ministerium für Soziales, Gesundheit, Arbeit und Familie (TMSGFF)).

### 2.3. Methods—Questionnaire Survey and Involvement of Local Health Authorities

As part of the CoMoTH project, a survey has been conducted to assess the needs and framework conditions of the public health service in Thuringia. In May 2022, the following questionnaire (see Table 1) has been mailed to all 22 local health authorities in Thuringia. The closed questions have been analyzed quantitatively using Excel, and the results have been presented descriptively in the form of bar charts. The free-text questions were evaluated qualitatively.

Furthermore, we have conducted online exchange rounds with the local health authorities from Thuringia. The state-level authorities, particularly the TLVW, participated in these activities and provided support with expertise. By the end of August 2022, two online exchange rounds had taken place. The following aspects were discussed at these events:

- presentation of the background and structure of the CoMoTH project;
- presentation of some fundamental aspects of wastewater monitoring and the analytical approach;
- exemplary presentation of interim results from CoMoTH;
- current developments at federal level and activities at EU level;
- discussion with the health authorities.

### 2.4. Methods—Logistics for Wastewater Sampling and Analytical Workflow

Between 27 December 2021, and 25 July 2022 (the end of the sampling period), up to 23 wastewater samples have been analyzed weekly. The samples have been collected as 24 h composite samples, typically during the night from Sunday to Monday. A 24 h composite sample consists of multiple sub-samples taken automatically at fixed intervals over 24 h to obtain a representative average wastewater sample [22,23,24,25,26]. For specific wastewater treatment plants (WWTP), sampling occurred on different days: at WWTP 19 from Wednesday to Thursday and at WWTP 18 from Thursday to Friday. From 21 March 2022, some of the wastewater treatment plants were sampled twice a week.

**Figure 4:**
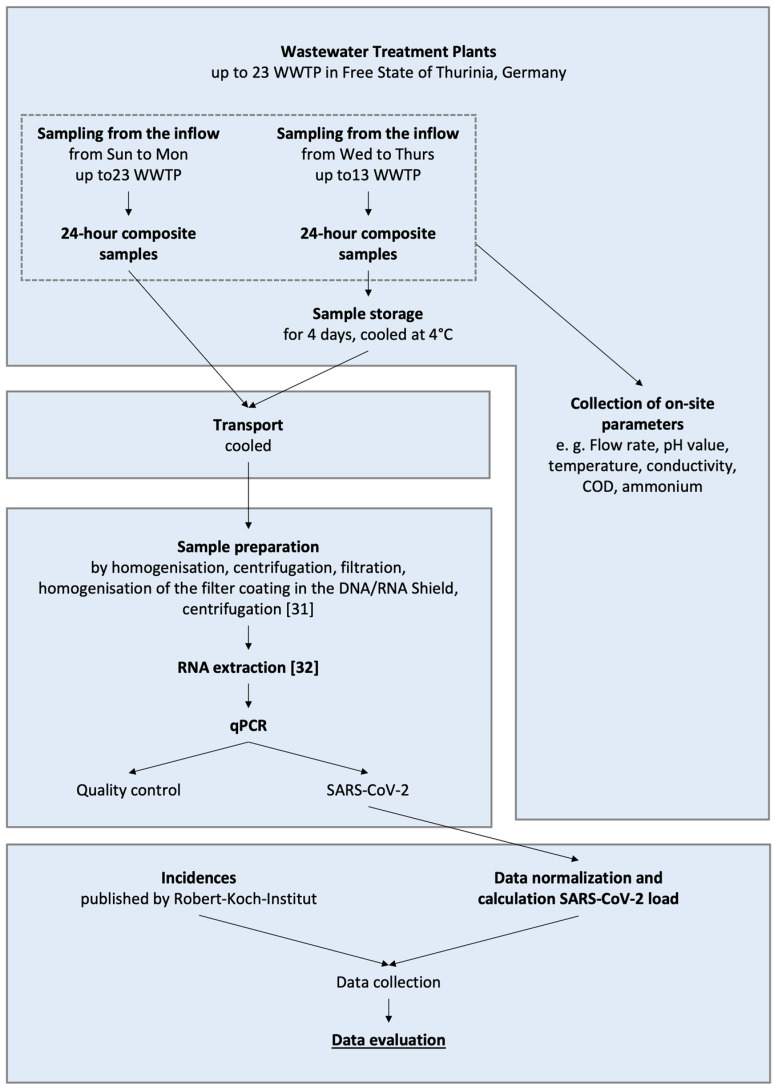
Sample logistics and workflow at BUW with sample preparation according to [31] and RNA extraction according to [32].

Figure 4 shows the analytical workflow and sample logistics. Sampling and the preparation of homogenized subsamples were conducted by trained personnel from wastewater treatment plant operators, following a set of clearly defined criteria. Each sample was documented using standardized sampling protocols. The samplers noted any deviations from the specified procedures or exceptional circumstances encountered during sampling. The completed sampling protocols have been collected at the Bauhaus University Weimar (BUW).

The 1000 mL samples from all WWTPs were transported once a week on Monday so that the samples, which were collected from Sunday to Monday, were transported to the laboratory on the same day. Samples collected on other days were kept refrigerated at 4 °C after sampling until the day of transport. Two different routes, a north, and a south route, were developed on the basis of the wastewater treatment plant locations, along which BUW employees collected the samples. The transport time for the longer route was around 7 h. During this time, the samples were stored in cool boxes at 4 °C. To minimize the risk of RNA degradation, the temperature of all wastewater samples was monitored after transportation. Published studies consistently show that SARS-CoV-2 RNA remains stable in raw wastewater under refrigerated conditions, and that cooling effectively preserves viral RNA integrity over typical sampling and transport intervals. This has been reported across multiple independent investigations [27,28,29,30]. The completed sampling protocols were transported along with the samples to the analytical laboratory of Bauhaus University Weimar (BUW). The sampling protocols were collected and digitized there.

Analytical workflow: Upon arrival at the wastewater laboratory, the 1000 mL samples were first homogenized by manual shaking to ensure an even distribution of the particles. Two separate aliquots of 50 mL each were then taken from each sample and centrifuged at 4000× *g* for two minutes.

The following workflow is based on the workflow developed by Analytik Jena [31], except for minor differences. The sample was concentrated by filtration. The filtration unit (Sartorius, Göttingen, Germany) was prepared with a membrane filter (HAWP04700, Merck, Darmstadt, Germany) made of mixed cellulose ester (MCE) with a pore size of 0.45 μm. A total of 100 mL was filtered from both centrifuge tubes of the same sample (50 mL each), taking care not to include the pellet. Filtration was carried out under a pressure of up to 8 bar. The loaded filter was removed and cut into four strips of equal width, and two strips were placed in innuSPEED Lysis Tubes (845-CS-1120100, Analytik Jena, Jena, Germany) with three metal beads. Two further metal beads were then placed on the filter strips before 1000 μL DNA/RNA Shield (ZYM-R1100-250, Zymo Research, Irvine, CA, USA) was added and the tubes were tightly sealed.

For the extraction negative control 100 mL tap water was filtered. The filter was divided into two lysis tubes, which were filled with phosphate-buffered saline (5418.0012, Chemsolute, Renningen, Germany) instead of DNA/RNA Shield.

Between the filtration runs, the filtration unit was rinsed and disinfected. The samples were then physically homogenized (SpeedMill Plus, 845-00007-2, Analytik Jena) for two minutes to disperse the filter coating in the solution. After homogenisation, the samples were stored at 6 °C in the refrigerator until extraction.

The samples were centrifuged (Biofuge pico, Heraeus, Hanau, Germany) for 2 min at 10,000× *g*. Nucleic acid extraction was performed with the InnuPure C16 touch (845-00020-2, Analytik Jena) using the innuPREP AniPath DNA/RNA Kit—ICP16 (845-IPP-8016096, IST Innuscreen GmbH, Berlin, Germany). For the extraction, 400 μL of the supernatant was taken from each lysis tube. The wells were filled according to protocol 2: Isolation from 400 μL cell-free body fluids [32]. After completion of the extraction, the two extracts of the same sample were combined.

According to the manufacturer’s instructions, the qPCR analysis was performed using the Water SARS-CoV-2 RT-PCR Test Kit (IDEXX, Westbrook, ME, USA). The kit was thawed for 15–30 min before use. Finally, the qPCR analysis was performed with the qTOWER^3^ (844-0055-2, Analytik Jena).

Consistent application of laboratory QA/QC measures ensured analytical reliability across sampling dates and wastewater treatment plants. Three positive controls, two no template controls (NTC) (with DNA/RNA-free water) and the extraction negative control were added to each well plate. the IDEXX assay’s internal amplification controls were used to identify potential PCR inhibition. Process controls accompanying filtration and extraction were also employed to verify consistent work-flow performance. The analytical sensitivity adhered to the validated LOD/LOQ specifications of the IDEXX Water SARS-CoV-2 RT-PCR Test Kit.

We calculated the virus concentration and viral load per inhabitant and day for each WWTP. Viral load was calculated as genome copies per inhabitant per day, combining measured SARS-CoV-2 concentrations with WWTP flow data and estimated population size. This metric enables comparisons across WWTPs of different sizes and provides a standardized indicator of infection dynamics within the catchment population [26,33,34]. As a state-wide indicator for the virus load, we pooled data from all WWTPs in the monitoring program, as described in Haeusser et al. [18].

### 2.5. Methods—Data Processing and Analytical Visualization

The success of wastewater surveillance systems hinges on the seamless integration, processing, and analysis of diverse data sources to generate actionable insights. An integrated approach towards data processing and visualization has been implemented, addressing the complexity of managing heterogeneous datasets and providing stakeholders with intuitive analytical tools. The conceptual design builds upon preliminary work conducted by the authors, described in [35,36]. This section presents the methodologies employed, the tools developed, and the challenges overcome during the project, subdivided into “data processing” and “analytical visualization”.

As described above, for appropriate data processing, the project has leveraged multiple datasets, including viral concentrations, water quality parameters, mobility data, meteorological information, and demographic statistics. The datasets have been processed using a structured framework to ensure consistency, accuracy, and relevance, as follows.

- Data cleaning and standardization: PCR results from wastewater samples have been standardized using Python-based automation using the Python 3.9 version scripts developed within a Jupyter environment, aiming to minimize manual errors and to aligned the data with standardized templates required by the Robert Koch Institute and the Federal Environment Agency. Water quality parameters, such as pH, temperature, and conductivity have been normalized to account for variability across different sampling sites. Missing values were imputed using the k-Nearest Neighbors (kNN) algorithm, ensuring a consistent dataset for further analysis. Mobility data sourced from anonymized cell phone signals and weather conditions (e.g., rainfall, UV index) have been harmonized to align temporally and spatially with the viral and water quality datasets. The dataset was split chronologically—January to August for training, September to November for testing, and December for validation—to prevent data leakage. Leakage was further minimized by fitting the entire data-processing pipeline, including standardization and kNN-imputation, exclusively on the training set before applying it to the test and validation data.
- Data integration and management: A centralized database integrating structured relational models and time-series storage systems (here, InfluxDB) has been established. Metadata, including geographic identifiers and sampling timestamps, have provided contextual relevance for cross-referencing diverse datasets. The pipeline also supports real-time data ingestion through Internet-of-Things-enabled sensor nodes [37], which transmit water quality metrics via MQTT protocols.

Regarding the analytical visualization, to translate complex data into actionable insights, a suite of visualization tools has been developed, tailored to the needs of the users (i.e., stakeholders). The tools combine advanced data analytics with intuitive interfaces to enhance decision-making. By merging technical precision with user-centric design, the tools facilitate the extraction of trends and patterns, enabling public health authorities and decision-makers to respond proactively, with focus emphasized on the following elements.

**Figure 5:**
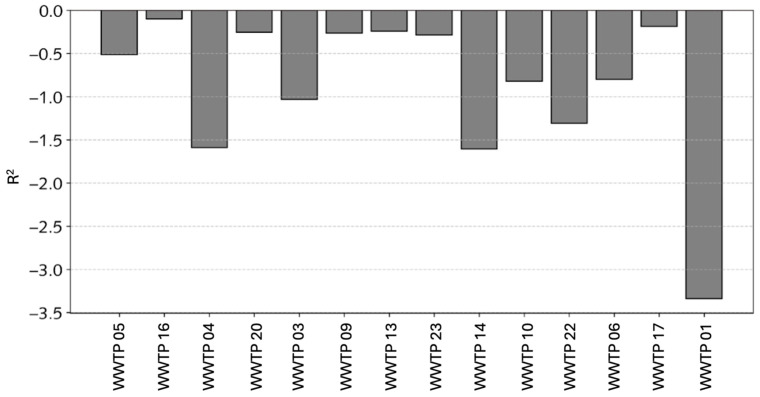
Performance evaluation of the decision tree AI model using the R^2^ value.

**Figure A1:**
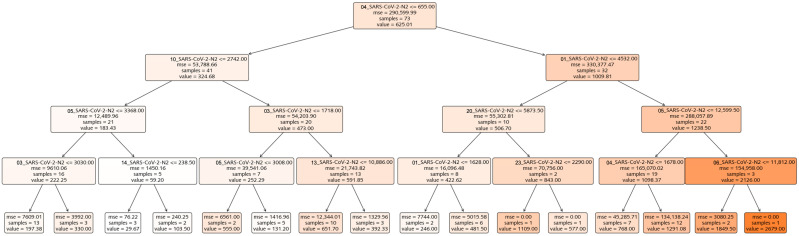
Example illustration of the decision tree for the wastewater treatment plant in WWTP 22 (tree depth = 4).

- GIS-based interactive dashboards: Interactive dashboards and GIS-based maps allowed for a granular analysis of spatial and temporal data, while predictive models integrated within the visualizations offered forecasts for epidemiological developments. The integration of artificial intelligence components further streamlines data exploration, simplifying complex analyses through natural language interfaces and automated visualization recommendations.
- A geographic information system (GIS) has been employed to visualize spatial data, such as viral load distributions and water quality parameters. Users may interact with layered maps to identify correlations between environmental factors and viral prevalence.
- Time-series analysis: Temporal trends in viral concentrations are represented through time-series plots, developed using Grafana. V11.1 The plots highlight, e.g., seasonal variations, mobility influences, and potential anomalies, providing a comprehensive overview of temporal dynamics.
- Digital twins and BIM integration: A digital twin is a virtual representation of a physical wastewater treatment facility, integrating structural, operational, and sensor data [38]. BIM (Building Information Modeling) provides the digital building data used to construct this virtual model [39,40]. Digital twins of wastewater treatment facilities have been developed based on building information modeling (BIM), following a generic digital twin reference architecture [41] and a well-established, generic BIM model [42]. The “virtual replicas” have integrated water quality and infrastructural data, enabling simulations of operational scenarios and predictive analyses.
- Augmented visualization and analytics using artificial intelligence: Various AI approaches have been employed to enhance the analysis and prediction capabilities in wastewater surveillance. Machine learning models have been developed and tested to predict viral concentrations and assess the correlations with environmental and demographic factors, such as support vector regression for handling non-linear patterns and decision tree regressors to capture hierarchical data structures. Ensemble methods, such as random forest regressors and gradient boosting regressors have also been utilized to improve prediction accuracy by combining multiple decision trees (See Appendix B, Figure A1 and Figure 5). Additionally, k-nearest neighbors regression has been applied for localized predictions, and multilayer perceptron models have been employed to capture complex patterns through neural networks. The AI models have been trained on the datasets integrating viral load, water quality, and mobility data, with the goal of identifying the most representative wastewater treatment plants and optimizing sampling strategies to reduce costs without compromising surveillance quality. The negative R^2^ values shown in Figure 5 indicate that the models cannot yet generalize well, primarily due to the limited amount of viral data available. The results should therefore be interpreted as a conceptual demonstration of what will be possible once larger, continuous datasets become available (e.g., through advanced sensor technologies or through larger data collection campaigns in WWTP), at which point the presented data pipeline and modeling approach are expected to gain practical relevance.

## 3. Results

### 3.1. Results—Workflow and Wastewater Monitoring

Within the project CoMoTH 839 wastewater samples were analyzed between 27 December 2021, and 25 July 2022. The workflow from sampling to qPCR-analysis was stable.

**Figure 6:**
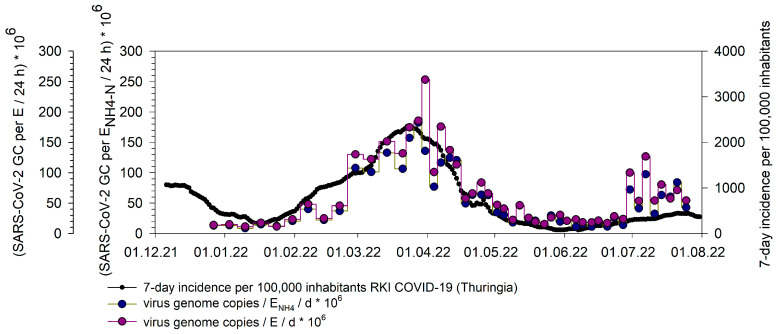
Timeline of SARS-CoV-2 genome copies per inhabitant (E)_NH4_/24 h and the SARS-CoV-2 genome copies per inhabitant (E)/24 h of sampled WWTPs (pooled dataset) and the 7-day incidence per 100,000 inhabitants for the Free State of Thuringia.

Figure 6 shows the timeline of pooled virus signals in the wastewater and the 7-day incidence on the federal level. The SARS-CoV-2 genome copies per inhabitant (E)_NH4_/24 h and the SARS-CoV-2 genome copies per inhabitant (E)/24 h of sampled wastewater treatment plants and the 7-day incidence for the Free State of Thuringia are visualized. From 21 March 2022, the samples were collected and analyzed twice a week at selected wastewater treatment plants. The normalization of the raw data was carried out according to the procedure described in Haeusser et al. [18].

To improve the reliability of viral load assessments in wastewater, normalization parameters are commonly used to compensate for fluctuations in the influent. In our work, we selected NH_4_-N as chemical population marker that are already established in wastewater-based monitoring. NH_4_-N is frequently highlighted in the literature [43,44,45,46]. It is a common approach in wastewater-based epidemiology, because ammonium nitrogen concentrations correlate with the human metabolic load and wastewater strength, thereby helping adjust viral concentrations for dilution effects [44,47,48]. NH_4_-N offers practical advantages, as this parameter is routinely measured directly at wastewater treatment plants and can be determined using inexpensive analytical methods. It is also recognized as an easily obtainable indicator that reflects the overall complexity of wastewater pollution. The ammonium concentrations of the samples for the WWTPs examined are given with a median value of 11 to 59 mg/L and a mean value of 11 to 59 mg/L [18]. The average values of the chemical parameter observed in our study are consistent with the typical concentrations reported for untreated municipal wastewater in Germany [49].

The time courses of the normalized pooled average concentrations of SARS-CoV-2 RNA values with NH_4_-N and population-based show similar trends. The infection wave during spring 2022 was distinctly observable from early February. From June onwards, a marked increase in the pooled data normalized using both NH_4_-N and population-based methods was observed. This discrepancy could be due to the change in Thuringia’s testing strategy: While asymptomatic individuals were eligible for free antigen testing until 29 June 2022 the group of eligible individuals was significantly restricted from 30 June 2022 [50]. This policy change might have reduced the coverage of asymptomatic infections in the 7-day incidences and a decline in the number of people tested, while wastewater monitoring continued to cover all infected groups.

### 3.2. Results—Sample Logistics and Laboratory Workflow

The entire sample logistics and analytical workflow could be implemented as planned, although complex process steps required particularly foresighted planning in order to meet deadlines and quality standards. This applied in particular:

- **Transportation time:** Due to the rural structure of Thuringia and narrow road conditions, sample collection on the north and south routes regularly exceeded the planned 7 h (Ø 8 h). Nevertheless, the parallel collection in both areas ensured that all samples arrived at the Bauhaus-Universität Weimar laboratory within 24 h.
- **Cold chain:** To minimize RNA degradation, samples could be kept refrigerated during transport and interim storage. Special care was required during the transfer processes and during the summer months.
- **Personnel effort:** The weekly coordination of transportation, documentation and sample distribution required considerable personnel resources, especially after the introduction of bi-weekly sampling from March 2022.
- **Time pressure:** Due to the large number of samples, homogenization, filtration and extraction had to be routine and standardized. Delays due to slow filtration steps (caused by interfering substances such as particles in the wastewater) required dynamic adaptation of the workflows.

Data exclusions: A total of 85 samples (≈9% of the dataset) were excluded from the analysis due to protocol deviations (e.g., defective automated sampler), measurement errors or unclear documentation in order to ensure data quality.

### 3.3. Results—Questionnaire Survey at Local Health Authorities

**Figure 7:**
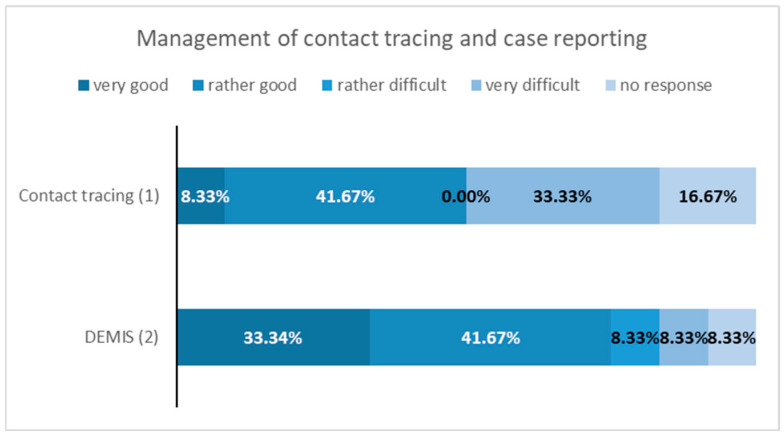
Results of the survey on questions 1 “contact tracing” and 2 “case reporting via DEMIS”.

**Figure 8:**
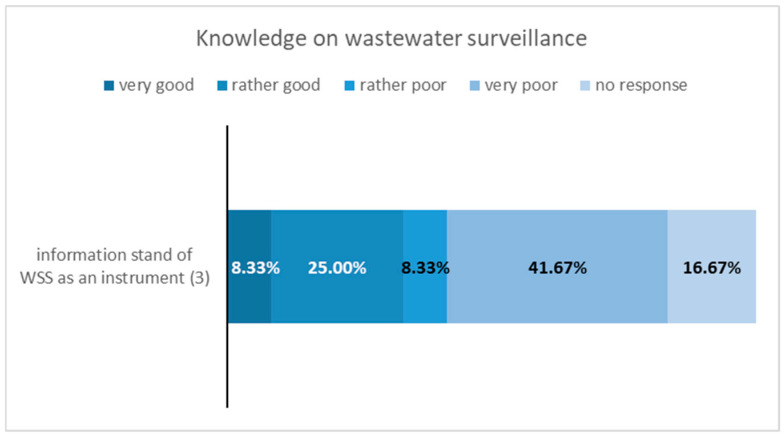
Results of the survey on question 3 “Knowledge on wastewater surveillance”.

**Figure 9:**
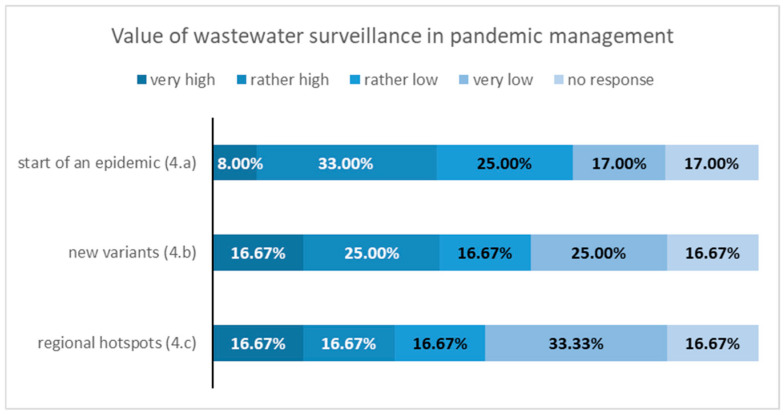
Results of the survey on question 4 “Value of wastewater surveillance in pandemic management”.

**Figure 10:**
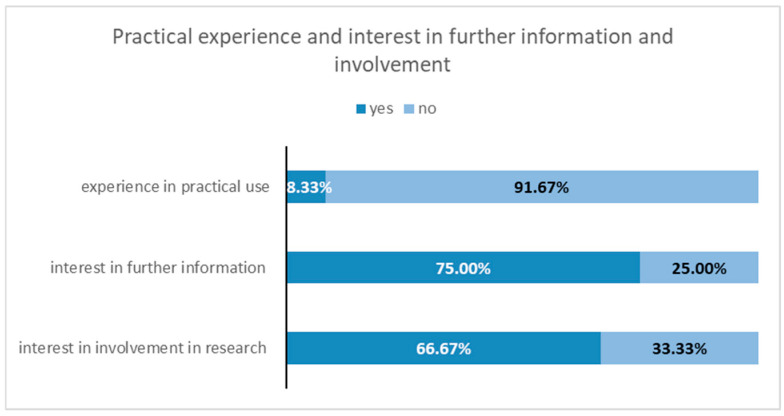
Results of the survey on question 7, 8 and 9 “Practical experience and interest in further information and involvement”.

The response rate for the questionnaire survey was 54%, with 12 out of 22 health authorities participating. Among the 17 districts and cities with at least one municipal wastewater treatment plant sampled for the CoMoTH project, the response rate was higher at 65%, with 11 out of 17 health authorities responding. The results of the questionnaire survey are illustrated in Figure 7, Figure 8, Figure 9 and Figure 10.

In May 2022 (time of the survey) more than one-third of the participating public health authorities have rated the contact tracing of cases as difficult. Reporting of cases using the reporting software (German Electronic Reporting and Information System for Infection Protection (DEMIS 1.0)) was “rather good” or “very good” according to seventy-five percent of participating health authorities.

Only thirty-three percent of the authorities assessed their knowledge of wastewater surveillance as “rather good” or “very good” and 50 percent as either “rather poor” or “very poor.”

Less than half of public health authorities see significant value in using wastewater surveillance for pandemic management. Specifically, over 40 percent believe that wastewater surveillance is helpful in the early stages of a pandemic and in detecting new variants. Additionally, 33 percent of these authorities rated the effectiveness of wastewater surveillance for identifying regional hotspots as either “very good” or “good.”

When asked about problems and challenges regarding implementation (question 5), the following aspects were mentioned frequently:

- Development of recommendations for action and guidelines for transferring the measured values from wastewater to risk management and measures to combat the pandemic—here, the desire for support from higher health authorities was mentioned (federal state/federal government);
- Legal basis for the initiation of measures;
- Support from political decision-makers;
- Clarified funding;
- Information and exchange.

Only one of the surveyed health authorities reported having experience in the practical use of wastewater surveillance. Most local public health authorities (75 percent) have expressed interest in obtaining more information, and 66 percent are interested in participating in future research projects (see Figure 10).

### 3.4. Results—Data Processing and Analytical Visualization

The data processing methodologies described in the previous sections have successfully generated manageable datasets, seamlessly prepared for data visualization. A critical initial step was data cleaning, which proved indispensable as raw data lacked the consistency and quality necessary for further processing. Automation scripts played a pivotal role by standardizing and aligning the datasets with templates required by authorities, ensuring accuracy and compliance.

For visualizing the analytical results, GIS-based interactive dashboards have become a standard approach in wastewater surveillance, being user-friendly and accessible to both experts and non-experts, eliminating the need for extensive training or instruction. In addition, the pre-existing GIS data for Thuringia simplifies cartographic work, as this foundational data is accessible via Al-Hakim et al., 2024 [36]. In CoMoTH, an interactive dashboard was implemented, which highlights counties and districts, enabling stakeholders to explore spatial data dynamically.

Artificial intelligence approaches, such as machine learning models, enhance the capability to detect patterns and predict trends. For instance, decision trees and ensemble methods were employed to analyze correlations between environmental factors and viral loads, while time-series analyses help identify temporal trends. The combination of these data processing and visualization methodologies provides a robust framework for stakeholders to access and interpret results tailored to their specific needs. The diversity of visualization methods, ranging from GIS dashboards to AI-driven analytics, enriches the surveillance system and enhances its value as a decision-support tool within CoMoTH.

## 4. Discussion

In this pilot study, we successfully monitored the viral level of SARS-CoV-2 in 23 wastewater treatment plants in a federal state in Germany. With our approach, we have captured the wastewater of more than 30% of Thuringia’s population. Over ten months, we established a foundation for developing a state-wide wastewater monitoring system using a bottom-up approach that engaged local stakeholders from both the health and the wastewater sectors. This study also included small and middle-sized wastewater treatment plants, which sets it apart from other monitoring programs. Special care was taken to ensure the sampling sites were distributed representatively across the state.

The structures and collaborations established in CoMoTH have been utilized in a follow-up project at the federal level. In the Abwassersurveillance TH project [51], the monitoring system was further developed and expanded to include monitoring of the influenza virus. Furthermore, some of the WWTPs participating in CoMoTH are now included in the national wastewater surveillance program in Germany. However, we must acknowledge that several challenges need to be addressed in the future to enhance the measurable benefits of wastewater-based surveillance and ensure its ongoing use. These challenges include issues related to funding, political support, and the need for guidelines and recommendations to effectively translate wastewater monitoring results into risk management and pandemic management strategies [17]. In Germany, some of these issues have been tackled through two research projects at the EU and federal levels—ESICora (Systematic Surveillance of SARS-CoV-2 in Wastewater) and AMELAG (Wastewater Monitoring for Epidemiological Situation Assessment) [52,53,54]. From 2022 to 2024, four technical guidance documents have been developed and published by the RKI and UBA within the framework of AMELAG, focusing on wastewater sampling, molecular biological analysis, sample logistics, and data submission and processing [55]. These projects, among others, have significantly contributed to harmonizing methods and enhancing wastewater-based surveillance of SARS-CoV-2 and other pathogens such as the influenza virus.

Since 2025, the AMELAG network has continued with approximately 50 selected WWTPs across Germany, corresponding to an average of around three indicative wastewater treatment plants per federal state [56]. The data generated through AMELAG are published both in the weekly wastewater surveillance report of the RKI and on the publicly accessible Infection Radar of the Federal Ministry of Health, where they serve as an additional epidemiological indicator available to professionals and the general public [57].

One important goal of wastewater surveillance systems is to enable public health officials to effectively interpret the data and use it to inform community interventions. Our project was the first in the federal state of Thuringia to involve local health authorities in wastewater surveillance. Our survey revealed that the data’s effectiveness for local decision-making is still limited and that more than half of the participating local health authorities are critical of the value of wastewater monitoring to inform community health interventions. On the other hand, the project revealed that over 60% of the health authorities in Thuringia who participated in the survey—representing 37% of all health authorities in the region—are interested in continuing information exchange programs about wastewater monitoring at national and international levels and in participating in future wastewater monitoring projects. This is not the majority of local health authorities in our study region. These findings align with the results of [16], which indicated that interest in conducting wastewater surveillance was lower at the local level compared to the state level. The SARS-CoV-2 pandemic has demonstrated the limits of the established surveillance systems. This is particularly challenging at the local level, as illustrated by the results of our questionnaire survey.

In the current situation, there is no urgent need for population-wide protective measures against SARS-CoV-2. On the other hand, there are no guiding parameters to help interpret and transfer data from wastewater monitoring to risk management, which would be beneficial. Establishing these parameters could provide a framework for local governance in future pandemic situations. Long-term promotion and support are necessary to strengthen local structures and expertise. Regular meetings and exchange formats for local health authorities could be highly beneficial in this context. These gatherings would facilitate sharing insights and experiences related to wastewater surveillance, enhance collaboration among authorities, and provide a platform for discussing challenges and successes in pandemic management. By creating a structured environment for ongoing dialogue, local health authorities could develop actionable guidelines and recommendations for the effective implementation of wastewater monitoring practices. Additionally, such exchanges could help build a supportive network that fosters trust and collaboration between health authorities and wastewater departments.

We have to acknowledge that the results of our questionnaire might be influenced by response bias and that, in particular interested local health authorities took part. So, results might not be representative for that state- or the national level.

Another key aspect is the digitalization of wastewater monitoring processes. The existing electronic reporting and information systems for infection control in Germany still present several complex challenges that make it difficult to integrate wastewater monitoring on a local level as an additional tool on time.

Our study demonstrates that pragmatic approaches to software architecture are currently on hand. The architectures should (1) be compatible with the existing equipment of the health authorities or the responsible state authorities and (2) be flexible enough to implement future adaptations quickly.

The study does not yet provide a comprehensive economic evaluation of the wastewater surveillance process. Cost–benefit aspects are discussed qualitatively but not quantified. Future analyses should therefore include techno-economic modeling and life-cycle cost assessment, incorporating parameters such as sampling frequency, analytical throughput, logistics costs, and avoided health expenditures. Integrating approaches such as Multi-Criteria Decision Analysis (MCDA) or cost–benefit frameworks could support the identification of optimal trade-offs between system sensitivity, operational costs, and decision utility.

In light of the newly adopted EU Directive 2024/3019 [58] on Urban Wastewater Treatment, the role of wastewater surveillance will gain additional strategic and legal importance. Paragraph (34) of the directive explicitly calls upon Member States to establish structured dialogue and coordination between public health authorities and wastewater management entities, ensuring that surveillance systems are operationally and institutionally aligned. Furthermore, Member States are required to define lists of public health–relevant parameters, as well as the frequency and location of sampling, taking into account the recommendations of ECDC, HERA, and the WHO. These parameters include SARS-CoV-2 and its variants, poliovirus, influenza virus, newly emerging pathogens, and other relevant indicators for public health. The directive also mandates that, in the event of a health emergency, such parameters must be regularly monitored as part of national preparedness and response strategies, drawing upon the lessons learned from the COVID-19 pandemic and the implementation of Commission Recommendation (EU) 2021/472 [3].

In addition, paragraph (35) of the directive [58] recognizes the growing importance of antimicrobial resistance (AMR) within the “One Health” framework and explicitly links wastewater surveillance to the broader European Action Plan on AMR. This reinforces the need for cross-sectoral integration between environmental, health, and laboratory infrastructures. Future research should therefore expand the surveillance scope to include AMR markers and pharmaceutical residues, integrating them into the existing analytical and digital frameworks. Such an expansion would not only strengthen pandemic preparedness but also contribute to the EU’s long-term vision of environmental health intelligence.

Finally, the CoMoTH study highlights organizational and governance barriers but does not quantitatively assess process maturity or institutional readiness. Methods such as process capability analysis, stakeholder mapping, or socio-technical system modeling could be applied to evaluate transition readiness and identify efficiency bottlenecks in the collaboration between wastewater utilities, laboratories, and public health authorities. Embedding these approaches within the governance context outlined by the EU Directive 2024/3019 would enable a more data-driven and reproducible assessment of transformation processes, bridging the gap between technical feasibility, legal requirements, and sustainable institutional implementation.

## Figures and Tables

**Table 1:** Questions from the questionnaire sent to the health authorities.

1. How do you currently (time of the survey) assess the management of contact tracing for the SARS-CoV-2 pandemic in your area of responsibility? *					
☐ very good	☐ rather good	☐ rather difficult		☒ very difficult	☐ no response
2. How do you rate the transmission of SARS-CoV-2 case reports from the laboratories to your company via DEMIS **? The transmission works….					
☐ very good	☐ rather good	☐ rather difficult		☐ very difficult	☐ no response
3. How would you rate your level of information on “wastewater monitoring as a supplementary tool for SARS-CoV-2 surveillance”?					
☐ very good	☐ rather good	☐ rather poor		☐ very poor	☐ no response
4. How do you assess the added value of wastewater monitoring for SARS-CoV-2 for management in different pandemic phases at the local level?					
(a) Added value at the start of a pandemic (part of an early warning system)					
(b) Added value when new variants occur (part of an early warning system)					
(c) Added value in the identification of regional and local focal points of the disease in a pandemic wave (improved information situation at local level)					
☐ very high	☐ rather high	☐ rather low		☐ very low	☐ no response
5. What obstacles and problems do you currently see in implementing wastewater monitoring in Thuringia as an effective surveillance tool for combating the pandemic?					
Free-text field					
6. In your opinion, which measures and prerequisites are of central importance for successfully implementing wastewater monitoring in your area of responsibility or in Thuringia?					
Free-text field					
7. Do you already have experience with the practical use of wastewater monitoring of SARS-CoV-2 (or other biological markers) in your area of responsibility?					
☐ yes			☐ no		
8. Is your health authority currently interested in an information event on wastewater surveillance?					
☐ yes			☐ no		
9. Is your health authority currently interested in actively participating in further research projects on wastewater surveillance?					
☐ yes			☐ no		

* additional free-text field to explain the reasons for possible difficulties; ** reporting software at local health departments.

## Data Availability

The data presented in this study are available on request from the corresponding author due to data protection regulations and privacy concerns (specify the reason for the restriction).

## Appendix A

**Table A1:** Sites and sampling points analyzed.

Site-ID	County LK/Urban District SK	Mean Flow Rate [m^3^ per d]	Size Classes	Population Served (Without Industry)
1	SK Weimar	16,747	4	66,500
2	LK Gotha	3539	4	8900
3	LK Ilm Kreis	6503	4	28,900
4	SK Gera	19,741	5	100,638
5	LK Ilm Kreis	8317	5	72,000
6	LK Schmalkalden-Meiningen	9509	4	30,000
7	LK Saale-Holzland-Kreis	2102	4	13,768
8	LK Saale-Orla-Kreis	4913	4	14,020
9	SK Jena	20,708	5	114,024
10	LK Nordhausen	9551	4	54,000
11	LK Eichsfeld	6115	4	14,358
12	LK Eichsfeld	2585	4	11,103
13	LK Eichsfeld	4689	4	55,867
14	LK Altenburger Land	1922	4	13,550
15	LK Soemmerda	3941	4	17,000
16	SK Erfurt	45,522	5	317,274
17	SK Suhl	17,872	4	36,000
18	LK Unstrut-Hainich-Kreis	2595	3	4569
19	LK Saale-Orla-Kreis	2335	3	5400
20	LK Schmalkalden-Meiningen	811	2	3500
21	LK Saalfeld-Rudolstadt	5154	4	28,817
22	LK Saalfeld-Rudolstadt	7651	4	32,808
23	LK Kyffhaeuserkreis	952	3	7000
